# Equity in maternal, newborn, and child health care coverage in India

**DOI:** 10.3402/gha.v6i0.22217

**Published:** 2013-09-10

**Authors:** Prashant Kumar Singh, Rajesh Kumar Rai, Chandan Kumar

**Affiliations:** 1 International Institute for Population Sciences, Deonar, Mumbai, India; 2 Tata Institute of Social Sciences, Deonar, Mumbai, India; 3 Department of Humanities and Social Sciences, Indian Institute of Technology Roorkee (IITR), Roorkee, Uttarakhand, India

**Keywords:** maternal, newborn and child health, composite coverage index, household economic status, states, India, millennium development goals

## Abstract

**Background:**

Addressing inequitable coverage of maternal and child health care services among different socioeconomic strata of population and across states is an important part of India's contemporary health program. This has wide implications for the achievement of the Millennium Development Goal targets.

**Objective:**

This paper assesses the inequity in coverage of maternal, newborn, and child health (MNCH) care services across household wealth quintiles in India and its states.

**Design:**

Utilizing the District Level Household and Facility Survey conducted during 2007–08, this paper has constructed a Composite Coverage Index (CCI) in MNCH care.

**Results:**

The mean overall coverage of 45% was estimated at the national level, ranging from 31% for the poorest to 60% for the wealthiest quintile. Moreover, a massive state-wise difference across wealth quintiles was observed in the mean overall CCI. Almost half of the Indian states and union territories recorded a ≤50% coverage in MNCH care services, which demands special attention.

**Conclusion:**

India needs focused efforts to address the inequity in coverage of health care services by recognising or defining underserved people and pursuing well-planned time-oriented health programs committed to ameliorate the present state of MNCH care.

India, with a population of over 1.21 billion, accounts for the highest number of maternal (estimated to be 56,000 in 2010) (1) and under-five deaths (estimated to be 1,655,000 in 2011) (2) in the world. The Millennium Declaration that was endorsed by the leaders from 189 countries in New York, USA, in 2000 also acknowledged the importance of regular monitoring and investigation in the progress and other dimensions of maternal and child health care through the fourth (reduce under-five mortality by two thirds between 1990 and 2015) and the fifth (reduce maternal mortality ratio by three quarters between 1990 and 2015) Millennium Development Goals (MDGs). India has been instrumental in devising new policies and programmes regularly as a response to unfavourable health outcomes, particularly in areas of maternal and child health care. However, despite the ambitious work-plan of several national level programmatic efforts such as the National Population Policy (2000), the National Health Policy (2002), and the National Rural Health Mission (2005), the progress in reducing maternal and child mortality is not satisfactory. Although a few regions or states of the country have shown considerable improvements and are on track to achieve the fourth and fifth MDG targets, the progress has been uneven and inequitable at several social, economic, and regional fronts. The increasing coverage of maternal, newborn, and child health care services is intrinsically associated with the expected improvements in the maternal and child health. Studies (3, 4) have shown that the increasing coverage gap (from the universal coverage) in maternal, newborn, and child health care services has considerable influence in under-five mortality in India. The sluggish progress in reducing maternal and child mortality in India could be attributed to the stark socioeconomic and regional inequality in the availability, accessibility, and affordability of healthcare services (4, 5). The WHO Commission on Social Determinants of Health (6) and the Rio-Political Declaration, 2011 (7), also highlight the importance of equity in achieving the goal of ‘Health for All’ (8). In this paper, we have portrayed the inequities in the coverage of maternal, newborn, and child health (MNCH) care services across five economic groups (i.e. wealth quintile) in India and its states using a Composite Coverage Index (CCI) (9). We have also discussed the challenges and implications for addressing health inequity in India's contemporary health agenda.

## Inequity in MNCH care coverage: definitions and measures

Inequity in health is a multidimensional concept (10), which is defined as inequalities in health that are unnecessary, avoidable, unfair, and unjust (11). Here, the inequity in MNCH coverage is presented in terms of absolute and relative inequalities. The absolute inequality is defined as the percentage coverage difference between economic groups, whereas the percentage coverage ratio between groups is a measure of relative inequality (5). The ‘Coverage’ is defined as the percentage of people receiving a specific intervention out of those who need it (12, 13). This is an important output of health care services and is regarded as an essential part of any strategy to monitor progress in program implementation (12).

Utilizing data from the third wave of the District Level Household and Facility Survey (DLHS-3) conducted during 2007–08 [International Institute for Population Sciences (IIPS) 2010], this study assessed the coverage in MNCH care services. The DLHS-3 was conducted by the IIPS under the aegis of the Ministry of Health and Family Welfare (MoHFW), Government of India. The IIPS ethical review board approved the survey procedures, and the MoHFW appointed the Technical Advisory Committee (TAC). A detailed description of sampling and data collection protocol is given in the DLHS-3 national report (14).

The CCI comprises a set of four intervention areas – family planning, maternal and newborn care, immunization, and treatment of sick children. The CCI has been developed to assess the coverage in MNCH care. A detailed description of the variables selected for constructing the CCI along with their definitions is presented in Table 1. The four selected subsets of intervention coverage indicators together represent all the stages of the continuum of care for reproductive, maternal, newborn, and child health, and this has been the major theme of the ‘Countdown to 2015 for Maternal, Newborn and Child Survival’ group (9). The CCI is calculated as:

CCI=0.25×(FPS+0.5×[SBA+ANCS]+0.25×[2DPT3+MSL+BCG]+0.5×[ORT+CPNM])

**Table 1:** Definition of indicators by intervention area used to construct the composite coverage index

Indicators for Composite Coverage Index	Definition
Indicators for family planning	
Need for family planning satisfied	Percentage of currently married women who say they do not want any more children or that they want to wait 2 or more years before having another child, and using contraception
Indicators for maternal and newborn care	
Skilled birth attendance	Percentage of live births in the 3 years before the survey attended by skilled health personnel (doctor, nurse, midwife, or auxiliary midwife)
Antenatal care	Percentage of women attended at least once during pregnancy by skilled health personnel for reasons related to the pregnancy in the 3 years preceding the survey
Indicators for immunisation	
Measles vaccination	Percentage of children aged 12–23 months who were immunised against measles
Diphtheria, Pertussis and Tetanus vaccination	Percentage of children aged 12–23 months who received three doses of Diphtheria, Pertussis, and Tetanus vaccine
BCG vaccination	Percentage of children aged 12–23 months currently vaccinated against BCG
Indicators for treatment of sick children	
Oral rehydration therapy	Percentage of children under-5 with diarrhoea in the preceding 2 weeks who received oral rehydration therapy (packets of oral rehydration salts, recommended home solution, or increased fluids) and continued feeding
Care seeking for pneumonia	Percentage of children aged 0–59 months with suspected pneumonia (cough and dyspnoea) who sought care from a health provider

where FPS is family planning need satisfied, SBA is skilled birth attendance, ANCS is antenatal care with skilled provider, DPT3 is three doses of Diphtheria, Pertussis, and Tetanus (DPT) vaccine, MSL is measles immunisation, ORT is oral rehydration therapy for children with diarrhoea, and CPNM is care seeking for pneumonia. The details of each variable are presented in Table 1. A Cronbach's α reliability coefficient (15) of 0.848 was estimated for the full set of eight coverage indicators suggesting high internal consistency among variables. Appropriate sample weight was applied to conduct the analysis. Analyses were performed using STATA version 10 (16) and Microsoft Excel.

In the absence of direct information on income or expenditure, the wealth index is considered as a robust surrogate measure of income at the household level (17–19). The DLHS-3 dataset provides a standard wealth index classified in five quintiles (Q1, Q2, Q3, Q4, and Q5), which is based on household amenities, assets, and durables. The principle of factor loading to amenities, assets, and durables derived by factor analysis is used for the computation of the wealth index (20). Households are categorised from the poorest to the wealthiest groups corresponding to the lowest to the highest quintiles, respectively. This study uses the term ‘wealth quintile’ and ‘economic groups’ interchangeably.

## Inequity in MNCH care coverage across wealth quintiles: a regional perspective

Table 2 shows regional differences in the mean coverage in MNCH care services in India and its states by wealth quintile using CCI during 2007–08. States from the north and the northeastern regions such as Bihar, Uttar Pradesh, Jharkhand, Madhya Pradesh, and Meghalaya along with another 10 states reported less than or approximately 50% coverage in MNCH care services. The mean overall coverage of 45% was estimated at the national level, ranging from 31% for the poorest to 60% for the wealthiest quintile. However, a massive state-wise difference was observed in the mean overall CCI, ranging from the highest for Goa (65%) to the lowest for Bihar (32%). The result shows that out of 28 states and union territories, the ratio of the mean coverage between the wealthiest and the poorest wealth quintile was about twice in almost 20 states. The MNCH coverage for the wealthiest group in several states such as Bihar, Uttar Pradesh, Jharkhand, Madhya Pradesh, and Chhattisgarh was, to some extent, observed identical to the MNCH coverage level for the poorest group in states like Goa, Tamil Nadu, and Kerala. The result also reveals that the ratio of the differences between the bottom two quintiles and the top two quintiles was well above 1.0 in eight states, indicating that the difference in MNCH care coverage between the top two (wealthiest and wealthier) wealth quintiles was relatively small compared to the difference between the bottom two (poorest and poorer) wealth quintiles.

**Table 2:** Composite Coverage Index (CCI) for maternal, newborn, and child health care services by wealth quintile and measures to describe equity for major states in India, 2007–08

		Coverage index across wealth groups					Measures to describe inequity				
			
States	Overall CCI	Poorest (Q1)	Q2	Middle (Q3)	Q4	Wealthiest (Q5)	Ratio (Q5/Q1)	Difference (Q5 − Q1)	(Q2 − Q1)/Q5 − Q4)
India	44.7	30.7	35.3	42.1	49.9	60.3	2.0	29.6	0.4
Bihar	31.6	24.1	28.2	33.9	43.3	55.6	2.3	31.5	0.3
Uttar Pradesh	35.0	26.1	28.8	32.9	39.0	52.3	2.0	26.2	0.2
Jharkhand	35.1	27.5	32.3	38.9	46.3	58.6	2.1	31.1	0.4
Madhya Pradesh	40.2	28.0	33.2	39.2	48.6	60.1	2.1	32.1	0.4
Meghalaya	40.3	30.5	33.3	39.5	48.3	59.3	1.9	28.9	0.3
Rajasthan	42.6	33.9	34.8	38.7	45.7	57.9	1.7	24.0	0.1
Chhattisgarh	42.7	34.7	39.4	44.0	50.5	58.6	1.7	23.8	0.6
Arunachal Pradesh	43.0	25.4	37.8	44.0	49.6	53.9	2.1	28.5	2.9
Uttarakhand	43.7	28.4	32.8	35.8	41.0	57.7	2.0	29.3	0.3
Assam	46.6	41.2	46.7	52.0	55.8	65.4	1.6	24.2	0.6
Gujarat	47.2	32.4	33.8	41.2	49.1	61.4	1.9	29.0	0.1
Mizoram	48.4	17.4	29.1	41.7	50.4	56.2	3.2	38.8	2.0
Manipur	48.4	30.4	41.5	52.6	58.7	65.8	2.2	35.4	1.6
Tripura	49.9	25.6	33.5	48.8	62.3	65.8	2.6	40.2	2.2
Orissa	50.3	41.9	49.2	55.5	60.9	64.1	1.5	22.2	2.2
Haryana	51.3	27.4	33.5	38.9	46.8	62.0	2.3	34.6	0.4
West Bengal	52.9	46.1	47.7	52.0	59.0	68.2	1.5	22.1	0.2
Maharashtra	53.1	41.4	48.0	50.7	55.3	62.0	1.5	20.7	1.0
Andhra Pradesh	53.4	40.5	47.3	50.9	55.4	58.9	1.5	18.4	2.0
Karnataka	53.7	44.2	48.8	50.9	56.9	63.6	1.4	19.5	0.7
Jammu & Kashmir	55.2	35.8	44.7	50.3	57.2	64.3	1.8	28.5	1.2
Himachal Pradesh	55.6	32.7	36.0	47.1	54.6	61.5	1.9	28.8	0.5
Sikkim	56.2	53.9	44.9	51.4	54.9	63.9	1.2	10.0	−1.0
Tamil Nadu	58.0	54.7	55.3	57.1	57.3	60.6	1.1	5.9	0.2
Kerala	59.7	52.4	49.5	57.4	59.7	60.5	1.2	8.1	−3.8
Punjab	60.0	31.3	41.3	42.7	50.9	66.6	2.1	35.3	0.6
Delhi	61.3	37.0	45.2	33.9	52.6	65.1	1.8	28.1	0.7
Goa	64.7	59.1	51.2	66.1	62.4	66.0	1.1	6.9	−2.2

## Challenges and implications

Undoubtedly, India has experienced considerable improvement in accelerating coverage in MNCH care since the Millennium Declaration, 2000. However, the persistent inequity in access to maternal and child health care across different economic groups is masked by the average improvement in the majority of states. An important hurdle in addressing this issue has been the identification of the deprived people who deserve special attention. Although different measures of economic inequity have consistently been portrayed across literature, India needs a standard measure to target the deprived in order to facilitate and encourage them to utilise maternal and child health care services.

Defining the poor in India is a sensitive issue that is often debated and it is indeed ironic that this hinders poverty alleviation efforts. For instance, the Lakdawala Committee (27.5% people live below the poverty line) and the Tendulkar Committee (37.2% people living below the poverty line) appointed by the Planning Commission for estimating the poverty line for 2004–05, have estimated two different poverty lines (21). In 2007, a report entitled, ‘Conditions of Work and Promotion of Livelihoods in the Unorganised Sector’ by the state-run National Commission for Enterprises in the Unorganised Sector (NCEUS) mentioned that 77% of the people lived below the expenditure on average of INR (Indian National Rupee) 20 per day per capita (22). The report also mentioned that most of those living below US$ 0.50 per day were from the informal labour sector with no job or social security, living in abject poverty (22). In 2012, the Planning Commission estimated that 29.8% of India's population lived below the poverty line (23).

There are evidences that about two-fifths of the Below Poverty Line (BPL) cards (one of the key eligibility criteria for availing governmental benefits) in India are with the non-poor households, and in several states, the majority of households in abject deprived groups do not possess BPL cards (24). Hence, in practice, there is no nationally acceptable standard measure to target the poor. The Organisation for Economic Co-operation and Development (OECD) report estimated that during the two decades up to 2008, the fall in the extent of absolute poverty was modest in India, compared to other countries such as Brazil, China, and Indonesia (25). However, the uncertainty in arriving at a standard measure of poverty affects the distribution of existing governmental health services benefits, which in turn adversely affects the people who are in utmost need and thus undermines India's efforts in reducing economic inequality.

The existing health inequity in India is due to the lack of attention to social determinants of health (including education, employment, improved water, hygiene and sanitation, nutrition, community and household environment, etc.) and the failure of the health system to provide essential health services to those in need (26). The low literacy levels among poor households coupled with a lack of proper health knowledge and health services/schemes are the key determinants of health care services utilization. It has been well acknowledged in Indian literature that poor households are socially marginalised, and indeed in many cases detached from the social network, which is one of the powerful tools for disseminating knowledge about the betterment of health among the marginalised sections of society. Moreover, lack of political commitment, vision, and programmatic initiatives based on the assessment of average performance/achievement of states rather than focusing on within and between groups inequity has made the vulnerable sections more disadvantaged. For many years, the low level of public financing at less than 1% of the total gross domestic product (GDP) along with wide variations across states is responsible for poor health coverage – for instance, Bihar's per capita expenditure is about three times lower than that of Kerala. India is lacking in many health infrastructure standards recommended by the World Health Organization (WHO), including the number of health workers per 10,000 population, which is 19 – well below the norms of 23.4 and the nurse-to-doctor ratio at 1.5:1 instead of the desirable 3:1 (27). According to Rural Health Statistics (2010), there is a shortage of 19,590 sub-centres, 4,252 PHCs and 2,115 CHCs in the country (28). Although, the Government of India agreed to raise public spending to over 1.5% of the total GDP during the Twelfth Five-Year Plan (2012–2017), it still requires the pooling of funds to meet the required health needs.

Low public spending on health has led to a high level of out-of-pocket expenditure, that is, about 78%, besides high hospitalization costs (23%), which make health services inaccessible to a significant proportion of Indian households (29). Recent estimates show that nearly one-fourth of the women in rural areas had not delivered their last baby in a health facility due to high costs (14). Evidences from developing countries have shown that out-of-pocket expenditure on health exacerbates poverty (30). In 2004–05, it was estimated that about 39 million Indian people fell into the poverty trap every year as a result of high out-of-pocket expenditure (29). The provisional results of the 68th National Sample Survey (July 2011–June 2012) on household consumer expenditure reported that in rural India, half of the population belonged to the households living with around INR 34, which is equivalent to about US$ 0.61 (median value) per day (31). This indicates that poor households’ need for health care can easily let them fall into catastrophic health expenditure, and eventually not able to escape from the poverty trap.

Given these circumstances, the poor suffer the most due to lack of sufficient money to access required health care. Although several schemes such as the *Rashtriya Swasthya Bima Yojana* (RSBY) and several other state-sponsored programs have attempted to increase access to health care, the impact of these schemes due to a shortage of health workers in primary health care facilities, along with issues related to poor governance and corruption, has come in the way against reducing out-of-pocket expenditure and equitable access to health care services (32). Moreover, primary health care is neglected in these schemes and public hospitals lag behind in benefiting from governmental funding for these programs. It has been recognised that programs and schemes designed only for the poor are more likely to end up with poor programs (26), in terms of design, implementation, and monitoring (33).

The Planning Commission, Government of India instituted a High Level Expert Group (HLEG) to prepare a roadmap for improving universal health coverage, which highlighted the equitable access to health services for all as well as public health services addressing the wider determinants of health (34). The HLEG has listed the expansion and augmentation of primary healthcare, strengthening of district hospitals, expansion and upgrading the skills of the health workforce, free provision of essential medicines, abolition of ‘user fees’, establishment of effective regulatory structures and support for active community participation as high priorities that require early action. Additionally, the HLEG has suggested the ‘National Health Package’ for essential health at the primary, secondary, as well as tertiary levels of care for all citizens of India by 2022. Hence, it is imperative to promote a comprehensive ‘package’, which will follow the line of ‘Continuum of Maternal, Newborn and Child health Care’ (35) to avert maternal and child deaths in India. Additionally, both centre and state government need to be involved in policy and program initiatives and in decision-making about who should receive priority, and how equity-promoting initiatives should be implemented (36).

To conclude, we recommend that sufficient efforts should be made to bring forth a standard measure to define poor households and conduct the targeted interventions among the poor sections of the society, addressing obstacles in the utilization of health care services irrespective of the state level average performance. It has been established that without addressing the marginalised sections of the society, particularly the poor in India and its states, the fourth and fifth targets of the MDGs cannot be achieved by 2015. From a study of the present pattern of existing health inequity in India and its states, it can be said that though effective measures have been taken, the fourth and fifth targets of the MDGs could be achieved only by some states and sub-group of population, but not by all.
